# Genetic predisposition meets cytokine imbalance: the influence of TNF-α (-308) polymorphism and TGF-β levels in pediatric acute lymphoblastic leukemia in Egypt

**DOI:** 10.1186/s12885-024-13224-3

**Published:** 2024-12-10

**Authors:** Roqaia E. Radwan, Wafaa M. El-kholy, Afaf Elsaed, Ahmad Darwish

**Affiliations:** 1 Physiology Section, Zoology Department, Faculty of Science, Mansoura University, Mansoura, Egypt; 2 Genetics Unit, Children Hospital, Mansoura University, Mansoura, Egypt; 3 Hematology, Oncology and Bone Marrow Transplantation Unit, Pediatric Department, Faculty of Medicine, Mansoura University, Mansoura, Egypt

**Keywords:** ALL, TNF-alpha, TGF-beta, SNPs, Cytokine levels

## Abstract

Evading apoptosis fuels the aggressive nature of acute lymphoblastic leukemia (ALL). This study explored the potential roles of TNF-α, a pro-apoptotic cytokine, and TGF-β, a pro-proliferative factor, in the risk of developing ALL in Egyptian children. We investigated the TNF-α rs1800629 polymorphism and serum TGF-β levels in 100 ALL patients and 100 healthy controls. Notably, specific variations in TNF-α (GA, AA genotypes, and dominant model) were associated with an increased risk of ALL, suggesting impaired apoptosis. Conversely, ALL patients exhibited significantly lower TGF-β levels, potentially promoting uncontrolled proliferation. Our findings suggest that lower TGF-β and the TNF-α (-308) dominant model are associated with an increased risk of ALL. Additionally, TGF-β demonstrated exceptional accuracy (AUC 0.995) as a potential marker, with 100% sensitivity and 96% specificity. These findings suggest that TNF-α and TGF-β may be associated with ALL susceptibility, though further research with larger and more diverse populations is necessary to confirm these results.

**Supplementary Information:**

The online version contains supplementary material available at 10.1186/s12885-024-13224-3.

## Introduction

Acute Lymphoblastic Leukemia (ALL) is the most prevalent pediatric malignancy, marked by the rapid proliferation of lymphoid progenitor cells within the bone marrow, disrupting normal hematopoiesis. It constitutes approximately 2% of all lymphoid malignancies in the U.S., with the highest incidence in children aged 2–5 years [1]. Stratifying patients based on relapse risk is crucial for optimizing treatment outcomes. Prognostic models like the Children's Oncology Group (COG) B-ALL algorithm utilize genetic, clinical, and minimal residual disease (MRD) parameters to guide therapeutic interventions [2].

The molecular etiology of ALL involves complex interactions between genetic predispositions and epigenetic modifications. Chromosomal aberrations and mutations disrupt critical cellular signaling pathways, driving uncontrolled proliferation of malignant lymphocytes [3]. Among genetic risk factors, single nucleotide polymorphisms (SNPs) are central to disease susceptibility, influencing gene expression and protein function. Identifying disease-associated SNPs is essential for enhancing diagnostic precision and therapeutic strategies [4]*.*

While established risk factors for ALL include prior infections, high birth weight, and environmental exposures, genetic syndromes such as Down syndrome and Fanconi anemia significantly elevate ALL risk due to compromised DNA repair mechanisms [5, 6]. For example, Down syndrome patients have a 20-fold increased risk of ALL, underscoring the role of genetic anomalies in disease pathogenesis.

Cytokines, particularly those involved in inflammation, are implicated in ALL pathogenesis. Elevated levels of pro-inflammatory cytokines such as tumor necrosis factor-alpha (TNF-α) have been associated with poor outcomes in leukemia [7]. The TNF-α (-308) polymorphism (rs1800629), a well-studied SNP, has been linked to increased TNF-α production and inflammatory states, potentially contributing to leukemogenesis [8]. Transforming Growth Factor Beta (TGF-β), a cytokine involved in cell growth and apoptosis, also plays a pivotal role in leukemia, where disrupted TGF-β signaling can contribute to disease progression. Serum TGF-β levels may serve as prognostic markers in leukemia [9].

In this study, we aim to investigate the role of TNF-α (-308) polymorphism and serum TGF-β levels in predicting ALL risk in Egyptian children. By examining these markers in 100 ALL patients and 100 healthy controls, we aim to identify potential genetic predispositions specific to the Egyptian population. Understanding these associations could enhance early diagnosis and provide insights into targeted therapeutic approaches.

Our research seeks to address a critical gap in pediatric leukemia diagnostics, particularly in underrepresented populations. The findings may contribute to global efforts in refining prognostic tools and developing more effective treatments for pediatric ALL, ultimately improving patient outcomes.

## Methods

### Study design and participants

This single-center case–control study was conducted to evaluate the relationship between TNF-α (-308 G > A) polymorphism and TGF-β levels in pediatric ALL patients. The study included 100 newly diagnosed pediatric ALL patients and 100 healthy, age- and sex-matched controls. Patients were recruited from the Pediatric Department at the Oncology Center, Mansoura University, between March 2021 and March 2022. The patient group included 64 males and 36 females, aged between 2 and 17 years (median age = 9.5 years). A stratified analysis of the cohort was performed, dividing patients into pediatric (2–10 years) and older children/adolescent (11–17 years) groups to address differences in ALL presentation across age groups.

The study aimed to explore whether the TNF-α (-308 G > A) polymorphism influences TGF-β levels, given the potential for cytokine cross-talk in cancer biology. While the relationship is indirect, the interaction between TNF-α and TGF-β plays a role in inflammation and immune regulation in the context of ALL. Future studies will include TGFB1 SNP analysis to clarify direct genetic influences on TGF-β expression.

### Control group selection

The control group consisted of 100 healthy children, matched by age and sex to the patient cohort, and from the same geographic region. These children were free from clinical signs of disease. Future studies will aim to include individuals with similar genetic backgrounds but without ALL-associated polymorphisms, to improve the precision of control comparisons.

### Sample size calculation

The sample size was determined based on the prevalence of the TNF-α (-308 G > A) polymorphism in the Egyptian population and the incidence of pediatric ALL. Sample size was calculated based on a power analysis. We anticipated a moderate effect size (Cohen’s d = 0.5) and set the significance level at 0.05, aiming for 80% power. Based on these assumptions, a minimum of 85 cases and 85 controls was necessary for the study. Previous studies in pediatric ALL were also considered to validate our sample size assumptions. Power analysis indicated that a sample of 100 patients and 100 controls would be sufficient to detect meaningful differences between groups. However, we recognize that the relatively small sample size may affect the statistical power, leading to variability in effect size estimations. Larger studies are necessary to validate our findings and improve generalizability.

### Inclusion and exclusion criteria

Inclusion criteria for the study included pediatric patients under the age of 18, newly diagnosed with ALL, and with no prior history of chemotherapy, radiotherapy, or chronic illness. Healthy controls were selected based on their absence of chronic illnesses, infections, or other conditions that might influence the study’s parameters, such as TGF-β levels. Exclusion criteria were rigorously enforced to ensure comparability between the patient and control cohorts.

### Clinical data collection

For all patients, clinical data including medical histories and examinations of liver, spleen, and lymph nodes were collected. Laboratory data, such as complete blood count, hemoglobin levels, platelet count, LDH levels, and the percentage of peripheral blood blasts, were documented. Blood samples for DNA extraction and serum biomarker analysis were collected at diagnosis, prior to any treatment, ensuring that baseline levels of cytokines and genetic markers were not influenced by therapy.

### DNA extraction and genotyping

Genomic DNA was extracted from peripheral blood samples using the Genejet™ Genomic DNA Purification Kit, following standard protocols as described by Bonin et al. [10]. DNA quality was confirmed using 1% agarose gel electrophoresis, stained with ethidium bromide, and visualized under UV light. DNA bands were photographed to document quality.

The TNF-α -308 G > A polymorphism was analyzed using the PCR-Amplification Refractory Mutation System (PCR-ARMS). The PCR conditions included an initial denaturation, followed by 35 cycles of denaturation, annealing, and extension to amplify the polymorphic regions. Allele-specific primers were used, and PCR products were separated by 2% agarose gel electrophoresis.

Specific TNF-α primers:

**Table:** 

TNF-α G forward primer:	5′-ATA GGT TTT GAG GGG CAT GG-3′
TNF-α A forward primer:	5′-AATA GGT TTT GAG GGG CAT GA-3′
Common reverse primer:	5′-TCT CGG TTT CTT CTC CAT CG-3′

### Serum TGF-β1 level measurement

Serum TGF-β1 levels were quantified using an ELISA (Boster’s Human TGF Beta 1 ELISA Kit), following the manufacturer's instructions and following the protocol established by Dong et al. [11]. Blood samples were collected via venipuncture, centrifuged to isolate serum, and stored at -20°C until analysis. The use of peripheral blood for cytokine measurement was justified due to ethical considerations surrounding bone marrow aspirations, which are more invasive and harder to justify for healthy control subjects. While bone marrow samples could provide more direct insights into the local leukemic microenvironment, previous studies have shown that peripheral blood cytokine levels can still provide valuable information about systemic immune dysregulation in ALL.

### Statistical analysis

Data were analyzed using SPSS software (Version 25.0), with results reported as mean ± standard deviation for continuous variables and as percentages for categorical variables. Genotype frequencies for TNF-α (-308 G > A) were compared between groups using chi-square tests, and Hardy–Weinberg equilibrium was assessed. Serum TGF-β levels were compared using Student’s t-test or the Mann–Whitney U test, depending on data normality. Age-stratified analyses were conducted to evaluate whether differences in cytokine levels and genotype distributions varied by age group, particularly between younger (2–10 years) and older (11–17 years) children. Logistic regression was used to assess the odds ratio (OR) of ALL risk in relation to TNF-α polymorphism and TGF-β levels, adjusting for potential confounders. Statistical significance was set at *p* < 0.05.

### Limitations and future directions

While the study provides valuable insights into the interplay between TNF-α polymorphism and TGF-β levels in pediatric ALL, we recognize its limitations. The indirect association between TNF-α SNP and TGF-β levels calls for further validation, including the investigation of TGFB1 polymorphisms. Additionally, larger sample sizes and the inclusion of bone marrow cytokine levels would enhance the robustness of our conclusions. Future research will also explore the relationship between these markers and clinical outcomes, such as drug resistance and treatment response, to provide a more comprehensive understanding of their role in ALL pathogenesis.

We acknowledge the potential limitations in using pre-treatment blood samples for genetic testing, given the risk of contamination with leukemic cells, which could compromise germline variant accuracy. However, we carefully selected cases with minimal leukemic cell burden in peripheral blood and implemented rigorous validation protocols to distinguish somatic mutations from germline variants. Future studies will consider remission-phase samples to confirm the findings and enhance the reliability of germline testing.

## Results

### Genetic studies

A genetic variation (SNP rs1800629) in the TNF alpha gene located on chromosome 6, is named TNFα − 308 G/A because it involves a Guanine (G) that can be replaced by Adenine (A) at the 308th nucleotide position. The G allele is more common (reference allele), while the A allele is less frequent (alternative allele). You can find more details about this specific SNP on the NCBI website using the provided ID, as shown in Supplementary Table 1 (S1).

### Hardy Weinberg equilibrium for studied SNPs

Assessing whether the TNF-α − 308 G/A (rs1800629) gene variant follows Hardy–Weinberg equilibrium in two groups: control (*n* = 100) and ALL (*n* = 100), likely representing patients with a specific disease, as shown in Table 1. The table shows the observed and expected genotype frequencies for GG, GA, and AA variants in each group. The analysis compares these frequencies and finds a good fit (*p*-values > 0.05) between observed and expected values in both groups. This result suggests that the genetic distribution of TNF-α − 308 G/A is stable within each group and is not influenced by external factors such as selection bias or non-random mating.

**Table 1:** Assessment of Hardy Weinberg equilibrium for TNF-α genotypes

*Genetic polymorphism*		*Control n* = *100*		*ALL n* = *100*	
		*Observed*	*Expected*	*Observed*	*Expected*
***TNFα −308 G/A (rs1800629)***	***GG***	*48*	*51.84*	*17*	*19.8*
	***AG***	*48*	*40.32*	*55*	*49.4*
	***AA***	*4*	*7.84*	*28*	*30.8*
	***p***	*0.163*		*0.525*	

The subjects were selected randomly from population in Dakahlia Governorate in Egypt. They were unrelated. Regarding rs1800629, among ALL group, 17 GG, 55 GA and 28 AA were observed, while among control group, 48 GG, 48 GA and 4 AA were observed.

### Baseline data

#### Demographic data and age range considerations

The study cohort comprised 100 individuals diagnosed with ALL with a mean age of 9.62 years, ranging from 2 to 17 years. This age distribution includes both the typical peak incidence age range of 2–5 years and extends into older children and adolescents. Table 2 provides a comparison of age and gender between the ALL group and the healthy control group.

**Table 2:** Comparison of age and gender among ALL patients and control groups

	*Control n = 100*	*ALL n = 100*	*Test (p)*
***Age (years)***			
* Mean* ± *SE*	*10.38* ± *0.35*	*9.62* ± *0.42*	*U* = *5516.0* *p* = *0.206*
* Median*	*10.0*	*10.0*	
* Range*	*2.0 – 17.0*	*1.0 – 17.0*	
***Gender***			
* Male*	*65 (65%)*	*64 (64%)*	*X*^*2*^ = *0.022* *p* = *0.883*
* Female*	*35 (35%)*	*36 (36%)*	

*SD* standard deviation, *SE* standard error, *min* minimum, *max* maximum, *X*^2^* chi square test*

Although the peak incidence of pediatric ALL is commonly observed between 2–5 years of age, our study also included older children up to 17 years. This broader age range was chosen to ensure a comprehensive understanding of ALL across different developmental stages. It is acknowledged that clinical presentations of ALL may differ between younger children and adolescents. However, the inclusion of older children in this study provides valuable insights into the potential variability of ALL presentation and progression in a broader age spectrum.

The distribution of gender within this group comprised 64% males and 36% females. Additionally, an equivalent number of 100 healthy control subjects were included, carefully matched for both age and gender with the ALL cases, as shown in Table 2. The age and gender distributions were carefully matched between the ALL cases and control subjects to minimize confounding variables. The statistical analysis confirmed that there were no significant differences in age or gender between the ALL cases and control subjects, ensuring that these factors did not bias the results.

By assessing the weight of the 100 ALL patients. The average patient weighs 31.54 kg (kg) with a standard deviation of 8.26 kg, indicating some variation around this value. Half the patients weigh more than 32.0 kg (median), while the other half weigh less. There's a total weight range of 32 kg (from 14.0 kg to 46.0 kg) within the ALL group, as shown in Table 3. Weight loss, a common clinical characteristic in many diseases, was investigated in the 100 ALL patients. As shown in Table 3, 61% (*n* = 61) of the patients did not report weight loss, while 39% (*n *= 39) did. The distribution of immunophenotypes in a group of 100 patients diagnosed with ALL is a laboratory technique used to identify specific cell surface proteins on leukemia cells, which helps classify the leukemia subtype. The immunophenotype in 100 ALL patients. Most (82%) have B-cell ALL, where leukemia originates from B lymphocytes, while 18% have T-cell ALL, arising from T lymphocytes. This distribution might reflect the overall higher prevalence of B-cell ALL or be specific to the study population, as shown in Table 3.

**Table 3:** Weight and immunophenotyping among ALL patients group

	*ALL n = 100*	
***Weight (kg)***		
* Mean* ± *SD*	*31.54* ± *8.26*	
* Median*	*32.0*	
* Range*	*14.0 – 46.0*	
	***Number***	***%***
***Weight Loss***		
* Absent*	*61*	*61.0*
* Present*	*39*	*39.0*
***Immunophenotyping***	***Number***	***%***
* B-ALL*	*82*	*82.0*
* T-ALL*	*18*	*18.0*

*SD* standard deviation, *SE* standard error, *min* minimum, *max* maximum

The blood cell profile of the studied 100 ALL patients reveals several key findings. Elevated white blood cell count (TLC): The average white blood cell count is significantly high (11.59 × 10^9/L) with a narrow range (9.00 – 12.92 × 10^9/L). Reduced red blood cell count (RBC), hemoglobin, and platelets: Compared to normal ranges, these values are lower on average (RBC: 3.79 × 10^12/L, Hemoglobin: 8.75 g/dL, Platelets: 78.93 × 10^9/L). Increased percentage of blasts: Both peripheral blood and bone marrow show elevated blast percentages, indicating the presence of immature leukemia cells (Peripheral: 26.87%, Bone marrow: 81.98%). The bone marrow has a wider range of blast percentages (44.00 – 105.00%) compared to peripheral blood, as shown in Supplementary Table 2.

The Rhesus factor (Rh) distribution among the 100 ALL patients shows that all 100 patients (100%) are Rh positive, and none were Rh negative, as shown in Supplementary Table 3. The study might have unknowingly recruited participants who are more likely to be Rh positive. This could happen if the recruitment process favored a specific geographic location or blood bank where Rh positivity is more prevalent. With only 100 participants, the study might not be large enough to capture the true distribution of Rh factors in the ALL population. A larger sample size would provide more statistically significant results. It's important to note that Rh factor itself is not known to be a risk factor for ALL. More research is needed to understand the reasons behind this observed distribution in this particular study.

### TNFα − 308 among studied groups

**Fig. 1:**
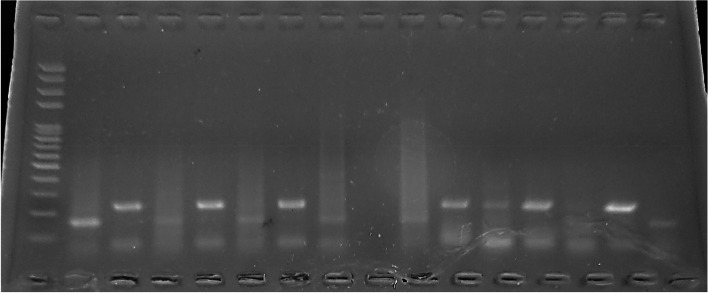
Agarose gel electrophoresis for TNF genotypes: ladder size marker (M) 50–1000 bp. Lane 1,3,5,7,9,13,15 A allele at 154 bp, Lane 2,4,6,10,11,12,14 G allele at 224 bp

TNF alpha gene polymorphism (− 308 G/A) was investigated using RFLP-PCR and classified as wild-type (GG), heterozygous carrier (GA), and homozygous variant (AA) genotypes. Results of PCR after gel electrophoresis are shown in Fig. 1.

Supplementary Fig. 1, 2 & 3 show agarose gel electrophoresis results for TNF genotypes in various exposure group.

**Fig. 2:**
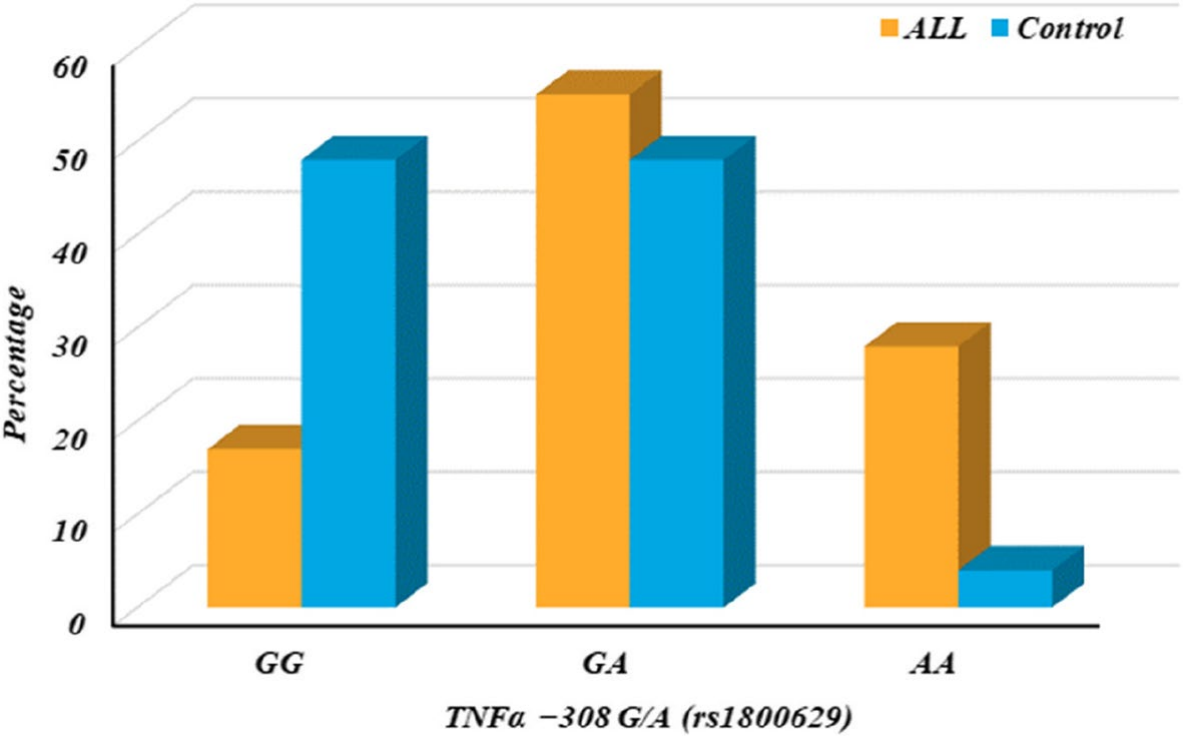
TNFα − 308 G/A (rs1800629) among ALL patients and control group

Investigating a variation in the TNF-alpha gene (rs1800629) between 100 ALL patients and 100 control subjects, the ALL group has a significantly higher frequency of the A allele (associated with variant AA and GA genotypes) compared to the control group (*p*-value < 0.001). This suggests a potential association between the TNF-alpha gene variation and ALL risk. Both dominant (GA + AA vs GG) and recessive (AA vs GG + GA) models show significant associations (*p*-value < 0.001). This means carrying either one or two copies of the A allele increases the risk of ALL compared to having only the G allele (considered the reference). The odds ratios (OR) further quantify this risk, with the AA genotype conferring the highest risk (OR = 5.983) compared to GG. The frequency of the A allele is substantially higher in the ALL group (55.5%) compared to controls (28.0%). This again suggests a potential role for this allele in ALL development, as shown in Table 4 and Fig. 2.

**Table 4:** TNFα − 308 G/A (rs1800629) among ALL patients and control group

*TNFα − 308 G/A (rs1800629)*		*Control n* = *100*		*ALL n* = *100*		*P value*	*OR (95% CI)*
		*N*	*%*	*N*	*%*		
***Genotypes***	***GG***	*48*	*48.0*	*17*	*17.0*	*-*	*Reference*
	***GA***	*48*	*48.0*	*55*	*55.0*	*0.001*	2.062 (1.371–3.102)
	***AA***	*4*	*4.0*	*28*	*28.0*	< *0.001*	5.983 (3.135*–*11.419)
***Dominant model***	***GG***	*48*	*48.0*	*17*	*17.0*	*-*	*Reference*
	***GA* + *AA***	*52*	*52.0*	*83*	*83.0*	< *0.001*	2.536 (1.713*–*3.754)
***Recessive model***	***GG* + *GA***	*96*	*96.0*	*72*	*72.0*	*-*	*Reference*
	***AA***	*4*	*4.0*	*28*	*28.0*	< *0.001*	3.782 (2.100*–*6.812)
***Alleles***	***G***	*144*	*72.0*	*89*	*44.5*	*-*	*Reference*
	***A***	*56*	*28.0*	*111*	*55.5*	< *0.001*	2.066 (1.600*–*2.668)

*N* number, *OR* odds ratio, *CI* confidence interval, Reference, according to NCBI database, *A* Arginine, *G* Glycine

*P*<0.05 is considered significant, OR<1 is considered protective, OR>1 is considered risky

### Association of TNFα − 308 with other parameters

This study of 100 ALL patients explored a potential link between the TNF-alpha gene variation (rs1800629) and weight loss, a common clinical presentation. Patients with the AA genotype (identified in Table 4 as the highest risk group) experienced weight loss at a higher proportion (42.9%) compared to those with GG (11.8%) or GA (45.5%). The TNF-alpha gene plays a role in inflammation, and the A allele might be associated with increased inflammation. This chronic inflammation could contribute to weight loss in some ALL patients. Notably, weight loss distribution differed significantly between rs1800629 genotypes (*p* = 0.040), with the highest incidence observed in the AA group, followed by GA and GG, as shown in Supplementary Table 4.

A variation in a specific TNFα gene (rs1800629) affects lab results in ALL patients as shown in Supplementary Table 5. The table divides patients into three groups based on their gene variation (GG, GA, AA) and shows various blood test results like platelet count, hemoglobin levels, and bone marrow blasts percentage. For most tests, there are no statistically significant differences (*p*-value > 0.05) between the genetic groups, meaning the gene variation likely doesn't influence those specific lab values in ALL patients.

The link between a TNFα gene variation (rs1800629) and the type of ALL in patients as shown in Supplementary Table 6. The table categorizes patients by their gene variation (GG, GA, AA) and shows the distribution of B-cell ALL (B-ALL) and T-cell ALL (T-ALL). While there's a trend of increasing T-ALL with more A alleles (GG to AA), the statistical test (*p*-value = 0.141) suggests that the IPT was not affected by rs1800629 genotypes (*p* > 0.05), and more data may be needed to confirm a definitive association.

The FAB subtypes (L1 and L2) are further delineated, and the percentage distribution of each genotype within these subtypes is provided. Notably, in FAB subtype L1, the GG genotype shows a higher prevalence (94.1%) compared to GA (56.4%) and AA (57.1%), emphasizing the potential role of this genetic variant in FAB subtype differentiation among ALL patients, as shown also in Supplementary Fig. 4.

### TGF-β among studied groups

**Fig. 3:**
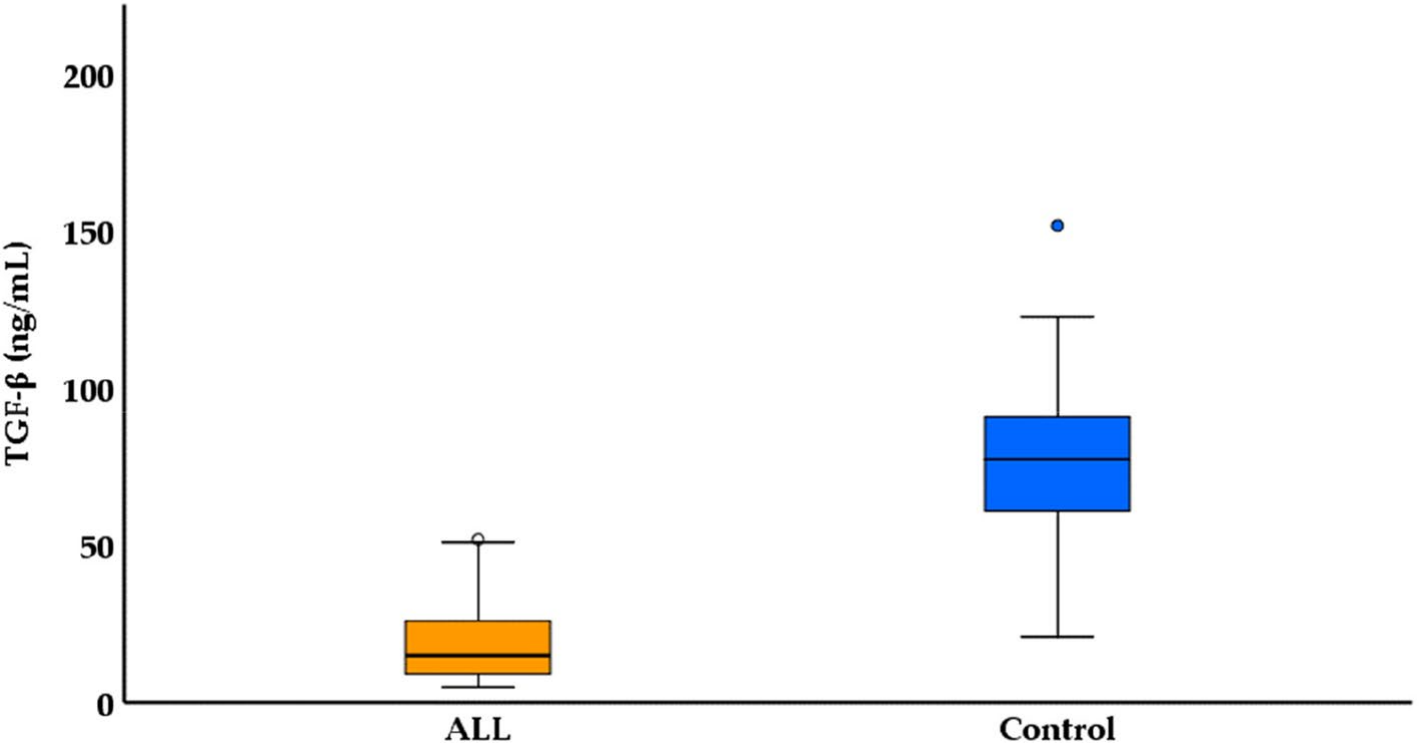
Boxplot for TGF-β among ALL patients and control group

A comparative analysis of Transforming Growth Factor-beta (TGF-β) levels between a control group (*n* = 100) and patients with ALL (*n* = 100). The TGF-β concentrations are provided in ng/mL, and the mean ± SE values for the control group (77.45 ± 2.03) and ALL patients (18.89 ± 1.22) highlight a substantial difference in TGF-β levels. A Mann–Whitney U test was conducted, resulting in a statistically significant *p*-value (*p* < 0.001), indicating a significant disparity in TGF-β concentrations between the two groups. The median TGF-β levels for the control group and ALL patients are reported as 77.50 and 15.0 ng/mL, respectively, further emphasizing the considerable reduction in TGF-β levels in ALL patients. The range of TGF-β concentrations in the control group spans from 21.0 to 152.0 ng/mL, while the range for ALL patients is narrower, ranging from 4.90 to 52.0 ng/mL. These findings suggest a potential association between TGF-β levels and the presence of ALL, underscoring the relevance of TGF-β in the context of leukemia pathophysiology, as shown in Table 5 and Fig. 3.

**Table 5:** Comparison of TGF-β among ALL patients and control group

	*Control n = 100*	*ALL n = 100*	*Test (p)*
***TGF-β (ng/mL)***			
* Mean* ± *SE.*	*77.45* ± *2.03*	*18.89* ± *1.22*	*U* = *9954.0* *p* < *0.001*
* Median*	*77.50*	*15.0*	
* Range*	*21.0 – 152.0*	*4.90 – 52.0*	

*SE* standard error, *min* minimum, *max* maximum. *U* Mann Whitney test

The association between the TNFα − 308 G/A (rs1800629) polymorphism and Transforming Growth Factor-beta (TGF-β) levels within the control group as shown in Supplementary Table 7. The table is organized based on the different genotypes (GG, AG, AA) of the TNFα − 308 G/A variant, and corresponding TGF-β concentrations are presented in ng/mL. The mean TGF-β levels for each genotype are reported as 73.96 (GG), 81.63 (AG), and 69.25 (AA). Although there is a numerical difference in mean TGF-β concentrations among the genotypes, the *p*-value (0.167) suggests that this difference is not statistically significant. The standard errors (SE) provide information on the variability of the mean estimates, and the medians, minimum, and maximum values offer insights into the distribution and range of TGF-β concentrations within each genotype. Overall, the table indicates that there is no significant association between the TNFα − 308 G/A polymorphism and TGF-β levels in the control group, as the *p*-value exceeds the conventional threshold of significance (*p* > 0.05).

**Fig. 4:**
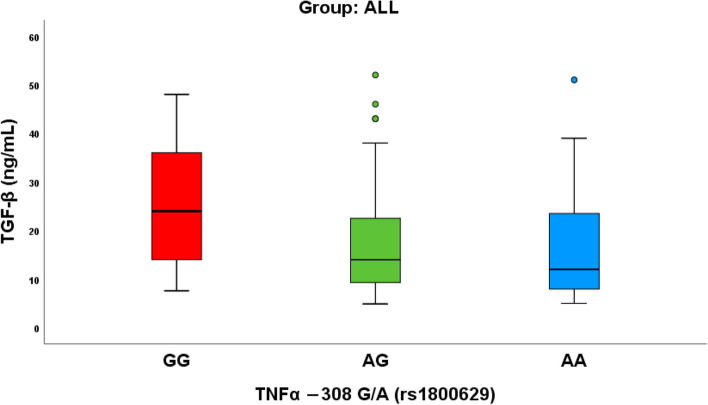
TGFB level between TNFα − 308 G/A (rs1800629) among ALL group

An analysis of the association between the TNFα − 308 G/A (rs1800629) polymorphism and TGF-β levels within the ALL group as shown in Supplementary Table 8 and Fig. 4. The table is structured by different genotypes (GG, AG, AA) of the TNFα − 308 G/A variant, and corresponding TGF-β concentrations are provided in ng/mL. The mean TGF-β levels for each genotype are reported as 25.68 (GG), 17.85 (AG), and 16.81 (AA). The *p*-value of 0.026 suggests a statistically significant association between the TNFα − 308 G/A polymorphism and TGF-β levels in the ALL group. Standard errors (SE) offer insights into the precision of the mean estimates, and medians, minimum, and maximum values provide information on the distribution and range of TGF-β concentrations within each genotype. Notably, the lower mean TGF-β levels in the AG and AA genotypes compared to GG suggest a potential impact of the TNFα − 308 G/A polymorphism on TGF-β regulation in the context of ALL, emphasizing the relevance of this genetic variant in leukemia pathophysiology.

### Prediction of ALL susceptibility

The providing results from regression analysis aimed at predicting susceptibility to ALL, as shown in Table 6. The table is divided into univariable and multivariable analyses, each with columns displaying *p*-values, odds ratios (OR), and their corresponding 95% confidence intervals (CI) for the variables TGF-β and TNFα − 308. In the univariable analysis, both TGF-β and TNFα − 308 show statistically significant associations with ALL susceptibility, as indicated by low *p*-values (< 0.001). The odds ratio for TGF-β is 0.891 with a 95% CI of 0.858–0.925, while TNFα − 308 has an odds ratio of 2.536 with a 95% CI of 1.713–3.754. In the multivariable analysis, both variables continue to exhibit significant associations with ALL susceptibility, with *p*-values < 0.001 for TGF-β and 0.014 for TNFα − 308. The odds ratios are adjusted to 0.997 (95% CI: 0.996–0.998) for TGF-β and 1.128 (95% CI: 1.090–1.268) for TNFα − 308. These findings suggest that TGF-β and TNFα − 308 are independent predictors of ALL susceptibility, emphasizing their potential roles in the development of the disease.

**Table 6:** Regression analysis for prediction of ALL susceptibility

	Univariable			Multivariable		
	*P*	OR	95% CI	*p*	OR	95% CI
**TGF-β**	< *0.001*	0.891	0.858**–**0.925	< *0.001*	0.997	0.996**–**0.998
***TNFα − 308***	< *0.001*	2.536	1.713**–**3.754	0.014	1.128	1.090**–**1.268

Logistic regression analysis was used

*OR* odds ratio, *CI* confidence interval

**Fig. 5:**
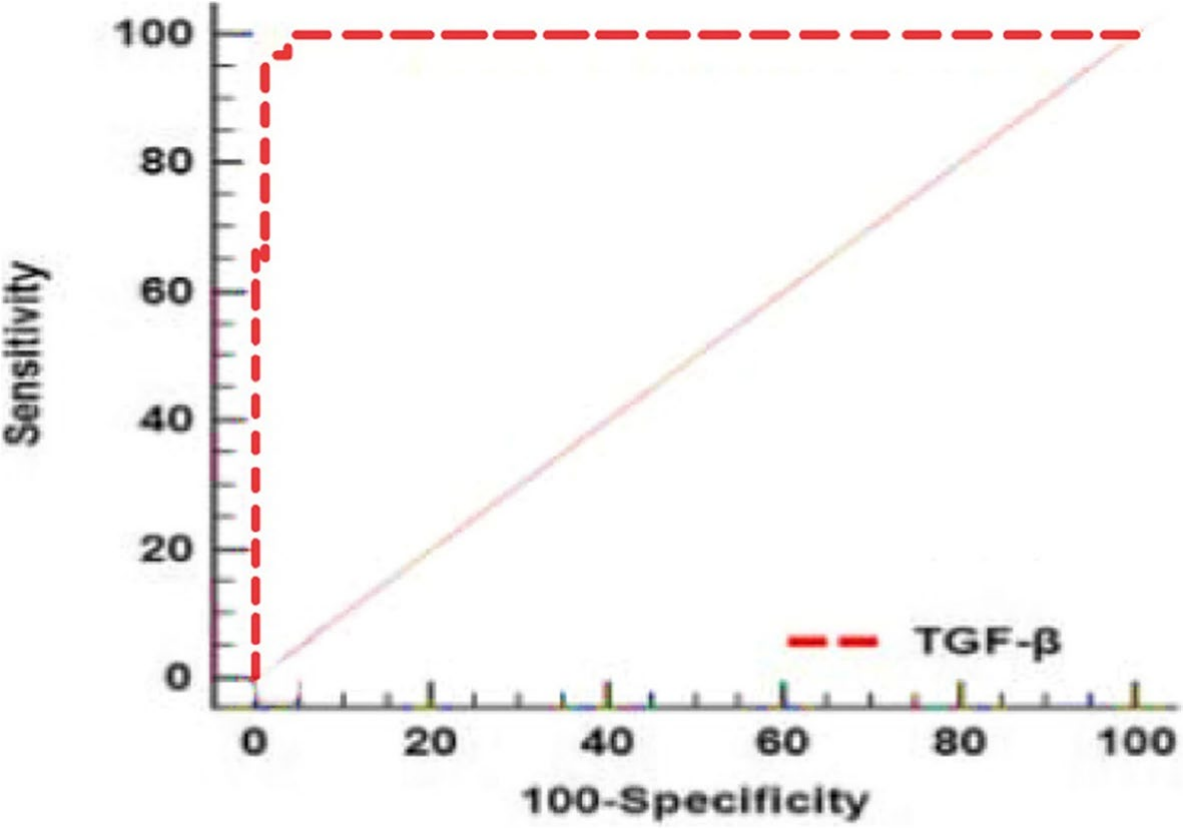
ROC of TGF-β for discrimination between ALL cases and control subjects

As illustrated in Table 7 and Fig. 5, TGF-β shows remarkable diagnostic accuracy for Acute Lymphoblastic Leukemia (ALL). Table 7 presents critical diagnostic parameters, including the Area Under the Curve (AUC), 95% Confidence Interval (CI), optimal cutoff value, sensitivity, and specificity. With an AUC of 0.995 within a 95% CI range of 0.973 to 1, TGF-β demonstrates an outstanding ability to differentiate ALL cases from controls. The optimal cutoff of ≤ 52 yields an impressive diagnostic performance, achieving 100% sensitivity and 96% specificity. These findings underscore the high reliability of TGF-β for accurately identifying ALL cases, particularly due to its exceptional sensitivity, which ensures true positives, and its high specificity, minimizing false positives.

**Table 7:** Validity of TGF-β for diagnostic ability of ALL

Variable	AUC	95% CI	Cut off	Sensitivity (%)	Specificity (%)
TGF-β	0.995	0.973—1	≤ 52	100	96

*AUC* area under ROC curve, *CI* confidence interval

While ALL diagnosis is conventionally achieved through routine hematologic and bone marrow assessments, the robust performance of TGF-β in our study highlights its potential role beyond primary diagnostics. The data support exploring TGF-β as a supplementary marker that could elucidate mechanisms in leukemia pathogenesis, contribute to prognostic assessments, and potentially guide targeted therapeutic interventions. This analysis substantiates TGF-β’s consistency and strength as a biomarker in clinical settings, suggesting its value not only in diagnostic applications but also as a promising avenue for future therapeutic and prognostic use.

The Receiver Operating Characteristic (ROC) curve for TGF-β reveals high diagnostic accuracy with an AUC of 0.995. The best cutoff value of 52 achieves 100% sensitivity and 96% specificity for distinguishing ALL cases from healthy controls.

## Discussion

### Acute lymphoblastic leukemia and hematologic profile

ALL is a hematologic malignancy that primarily impacts the hematopoietic system, predominantly affecting children and young adults. In our study, which included 100 pediatric ALL patients from Mansoura University Hospital, we aimed to identify biomarkers and genetic factors that may contribute to the pathogenesis of ALL. The hematologic profile observed in this cohort, with a mean total leukocyte count (TLC) of 11.59 ± 0.72 (× 10⁹/L), red blood cell (RBC) count of 3.79 ± 0.48 (× 10^12^/L), hemoglobin level of 7.5 ± 0.69 g/dL, platelet count of 78.93 ± 3.51 (× 10⁹/L), and bone marrow blasts at 81.98 ± 15.71%, aligns with findings from previous studies, supporting the validity of our patient cohort's clinical and hematologic characteristics [12].

### Immunophenotyping and FAB Classification

Immunophenotyping of our ALL cohort revealed a predominance of B-ALL (82%) compared to T-ALL (18%), which is consistent with the distribution observed in comparable studies [13]. This classification reflects the higher incidence of B-cell lineage involvement in pediatric ALL. The FAB (French-American-British) classification identified L1 morphology in 63% and L2 morphology in 37% of our patients, findings that align with previous reports by Kakaje et al. [14] and reinforce the demographic and morphological representativeness of our cohort.

### Role of TGF-β in ALL

Cytokines are pivotal regulators of immune responses and cellular behavior, exhibiting both pro- and anti-tumorigenic effects in cancer by influencing the tumor microenvironment, modulating immune surveillance, and affecting therapeutic outcomes, with recent advances in targeting cytokine and chemokine signaling pathways—such as monoclonal antibodies and receptor inhibitors—offering promising avenues for overcoming resistance and enhancing the efficacy of cancer treatments [15].

The interplay between TNF-α (-308) and TGF-β is complex, with TNF-α promoting inflammatory responses and TGF-β functioning as both a pro-inflammatory and regulatory cytokine depending on the context. Several studies have suggested that polymorphisms in TNF-α could affect cytokine signaling, influencing TGF-β activity indirectly through inflammatory cascades. While our findings suggest an association, we acknowledge that further studies are needed to fully elucidate this relationship.

Impairment in TGF-β-induced regulatory T (iTreg) cell differentiation significantly disrupts immune homeostasis and contributes to various autoimmune diseases, making therapeutic strategies aimed at enhancing iTreg differentiation increasingly relevant. In the study of Zhang et al., [16], they demonstrate that autoimmune inflammation in experimental autoimmune encephalomyelitis (EAE) is linked to selective impairment of iTreg differentiation primarily due to elevated TNF-α production. Notably, neutralizing TNF-α enhances iTreg differentiation and alleviates EAE symptoms, while depleting iTreg cells negates the efficacy of anti-TNF-α therapy. The findings reveal that TNF-α inhibits iTreg differentiation through a signaling cascade involving TNF receptor II (TNFR2) induction and Akt activation, which disrupts TGF-β-mediated Smad3 phosphorylation and subsequently reduces foxp3 transcription. Importantly, this regulatory pathway is specific to iTreg cells, as TNF-α does not activate Akt in naturally occurring regulatory T cells. These insights elucidate the critical mechanisms by which TNF-α regulates iTreg differentiation and support the therapeutic potential of TNF-α antagonists in treating autoimmune diseases.

In the context of our study on pediatric ALL, the balance between pro-inflammatory and anti-inflammatory cytokines plays a crucial role in disease progression and immune regulation. TNF-α, a pro-inflammatory cytokine, is known to promote inflammatory responses, while TGF-β can act both as a pro-inflammatory and regulatory cytokine depending on the immune environment [17].

According to a study of Dai et al., [18], immune dysregulation is increasingly recognized as a key factor in the pathogenesis of hematologic malignancies, yet limited research has explored cytokine alterations in childhood B-cell acute lymphoblastic leukemia (B-ALL) at diagnosis. This study aimed to characterize the cytokine network in newly diagnosed pediatric B-ALL patients by measuring serum levels of interleukins (IL)-2, IL-4, IL-6, IL-10, tumor necrosis factor (TNF), interferon (IFN)-γ, IL-17A, and transforming growth factor-β1 (TGF-β1). The study observed significant elevations in IL-6 (*p* < 0.001), IL-10 (*p* < 0.001), and IFN-γ (*p* = 0.023) in B-ALL patients, alongside a marked reduction in TGF-β1 (*p* = 0.001).

We observed significantly lower serum levels of TGF-β in ALL patients compared to healthy controls. While TGF-β is known for its dual roles in cancer, acting as both a suppressor and promoter depending on the context [19], our findings suggest its potential role as a tumor suppressor in early leukemogenesis. However, the observed TGF-β levels could also be a secondary effect of the disease rather than a direct cause. Future studies utilizing bone marrow aspirations might provide more accurate insights into TGF-β levels in the context of ALL [20, 21].

### TNF-α and genetic polymorphisms

We hypothesize that the TNF-α (-308) polymorphism may influence the inflammatory environment in children with ALL by altering TNF-α expression, thereby indirectly affecting TGF-β activity. This interaction may disturb the delicate balance between pro-inflammatory and regulatory cytokines, contributing to disease progression [17].

The study of Schober et al., [22] investigates the effects of key Th1 cytokines, TNF-α and IFN-γ (interferon gamma), on ALL cell lines and patient-derived xenografts (PDX). By analyzing their responses and correlating these with the expression of cytokine receptors and intracellular signaling molecules, the study reveals significant heterogeneity in cell death outcomes. Apoptosis emerges as the predominant form of cell death induced by Th1 cytokines in ALL cells. Interestingly, IFN-γ receptor (IFNGR) is expressed at higher levels than TNF receptors in the leukemia cells, leading to stronger phosphorylation of STAT1 (signal transducer and activator of transcription) relative to NF-κB (nuclear factor kappa-light-chain-enhancer of activated B-cells), which mediates TNF signaling. STAT1 activation correlates strongly with the extent of cell death following Th1 cytokine exposure.

Our study highlighted the role of TNF-α as a pro-inflammatory cytokine involved in ALL pathogenesis. Elevated TNF-α levels in our cohort are consistent with previous studies linking TNF-α to chronic inflammation and leukemogenesis [23, 24]. The TNF-α rs1800629 polymorphism has been associated with higher cytokine production and increased leukemia risk [8, 25]. Our findings align with Zhou et al. [26], who reported a positive correlation between elevated TNF-α levels and poor prognosis in leukemia patients.

Additionally, we acknowledge the absence of analysis on the TNF-α -238G/A polymorphism in our study. The -238G/A variant may also influence TNF-α expression and deserves consideration in future research to provide a more comprehensive understanding of TNF-α’s role in ALL susceptibility.

### Specificity of TNF-α polymorphisms to ALL over AML

An intriguing observation from our study is the association of TNF-α polymorphisms with ALL rather than Acute Myeloid Leukemia (AML). This specificity may be attributed to lineage-dependent differences in cytokine signaling pathways. Lymphoid progenitor cells, which give rise to ALL, may be more susceptible to the pro-inflammatory effects of TNF-α, leading to dysregulated proliferation and survival. In contrast, myeloid progenitors involved in AML may respond differently to TNF-α-mediated signals, possibly through distinct receptor distributions or downstream signaling mechanisms. The differential expression of TNF receptors and the subsequent activation of signaling cascades, such as NF-κB in lymphoid cells versus other pathways in myeloid cells, could underlie the selective predisposition to ALL. Further studies exploring these lineage-specific responses are essential to elucidate the mechanistic basis for this observation.

### Inflammatory cytokines and ALL pathogenesis

Cytokines, as critical molecular messengers, play multifaceted roles in cancer development by regulating both pro- and anti-tumor activities, including immune responses, inflammation, angiogenesis, tumor cell invasion, and metastasis, making them key targets for understanding cancer pathogenesis and developing novel therapeutic strategies [27].

The interplay between TNF-α and TGF-β in ALL underscores the complex role of cytokine signaling in leukemia. While TNF-α promotes inflammation and may exacerbate disease progression, TGF-β’s role appears to be more context-dependent, potentially exhibiting tumor-suppressive effects that are overridden in the leukemic microenvironment. This cytokine dysregulation highlights the potential for targeting these pathways in therapeutic strategies aimed at modulating the immune response in ALL [28].

The study by Whitehead et al. [29] further supports the notion that prenatal immune factors, such as elevated cytokine levels at birth, may predispose individuals to ALL by fostering an abnormal immune environment conducive to leukemic transformation. In this study, seven cytokines (IL1β, IL4, IL6, IL8, GM-CSF, TNFα, and VEGF) were measured in neonatal blood spots from 1,020 ALL cases and 1,003 controls in the California Childhood Leukemia Study. Higher levels of IL1β, IL8, TNFα, and VEGF at birth were associated with an increased risk of ALL, particularly in children of Latina mothers and those with high hyperdiploidy. Although neonatal cytokine levels correlated with endogenous metabolites previously linked to ALL risk, the cytokines did not mediate this relationship. These findings suggest that children born with elevated cytokine levels may be predisposed to abnormal immune responses, potentially initiating the development of ALL.

### Implications for therapeutic strategies

The dysregulation of cytokine signaling pathways in ALL presents potential targets for therapeutic intervention. Modulating TNF-α levels or its signaling pathways could mitigate the pro-inflammatory environment that supports leukemic cell survival and proliferation. Conversely, restoring TGF-β’s regulatory functions might re-establish immune homeostasis and inhibit leukemic progression. The therapeutic potential of TNF-α antagonists, as suggested by Zhang et al. [16], highlights the promise of targeting specific cytokines to enhance regulatory T cell differentiation and suppress leukemic inflammation.

## Conclusion

Our study contributes to the growing body of evidence that ALL pathogenesis is driven by a combination of genetic predispositions and cytokine dysregulation. The observed alterations in TGF-β and TNF-α levels, along with the presence of the TNF-α -308G/A polymorphism, provide valuable insights into the molecular mechanisms underlying ALL. These findings underscore the potential for targeting cytokine pathways in developing novel therapeutic strategies aimed at restoring immune balance and counteracting tumor-mediated immune suppression. Future research should explore the comprehensive genetic landscape, including additional polymorphisms such as TNF-α -238G/A, and investigate the therapeutic modulation of cytokines to enhance treatment efficacy and patient prognosis in ALL.

## Supplementary Information

Supplementary Material 1.

## Data Availability

The datasets used and/or analyzed during the current study are available from the corresponding author on reasonable request.

## Abbreviations

ALL: Acute Lymphoblastic Leukemia
SNPs: Single Nucleotide Polymorphisms
TNF: Tumor Necrosis Factor
TGF-β: Transforming Growth Factor Beta
AL: Acute Leukemia
REC: Research Ethics Committee
IRB: Institutional Review Board
PCR: Polymerase Chain Reaction
HWE: Hardy–Weinberg equilibrium
NCBI: National Center for Biotechnology Information
ROC: Receiver Operating Characteristic
ECM: Extracellular Matrix
EMT: Epithelial-Mesenchymal Transition
LAP: Latency Associated Peptide
NK: Natural Killer
