# Supplementary material for: Genetic predisposition meets cytokine imbalance: the influence of TNF-α (-308) polymorphism and TGF-β levels in pediatric acute lymphoblastic leukemia in Egypt
Source: BMC Cancer. 2024 Dec 10;24:1509. doi: 10.1186/s12885-024-13224-3 (PMC11629532; doi:10.1186/s12885-024-13224-3)
Supplement: Supplementary file 1 — Supplementary Material 1. [file 12885_2024_13224_MOESM1_ESM.pdf]

Supplementary Table 1:

S1. Genetic features of studied SNPs according to National Center for Biotechnology Information (NCBI).

**ID**	**TNFα −308 G/A**
	**rs1800629**
**Alleles**	GA
**Reference Allele**	G
**Alternative allele**	A
**Gene**	TNF
**Chromosome**	6
**Reference**	[***https://www.ncbi.nlm.nih.gov/snp/rs1800629***](https://www.ncbi.nlm.nih.gov/snp/rs1800629)

Alleles: This indicates the possible variations at this location. In this case, it can be either Guanine (G) or Adenine (A). **Reference Allele:** This is the most common allele in the population, denoted by G. **Alternative Allele:** This is the less common allele, denoted by A.

Supplementary Table 2:

S2. Haematological parameters among patients with ALL.

	ALL ***n = 100***
TLC ($X10^9/L$)	
Mean ± SD.	*11.59 ± 0.72*
Median (Range)	*11.60 (9.00 – 12.92)*
RBC ($X10^{12}/L$)	
Mean ± SD.	*3.79 ± 0.48*
Median (Range)	*4.00 (2.80 – 4.60)*
Hemoglobin (g/dL)	
Mean ± SD.	*8.75 ± 0.69*
Median (Range)	*8.80 (6.10 – 10.34)*
Platelet ($X10^9/L$)	
Mean ± SE.	*78.93 ± 3.51*

Median (Range)	*64.50 (45.00 – 157.00)*
Peripheral blasts (%)	
Mean ± SD.	*26.87 ± 4.48*
Median (Range)	*27.0 (20.0 – 35.0)*
Bone marrow blasts (%)	
Mean ± SD.	*81.98 ± 15.71*
Median (Range)	*86.50 (44.00 – 105.00)*

SD, standard deviation, SE, standard error; min, minimum; max, maximum.

Supplementary Table 3:

S3. Serologic data among patients with ALL.

	ALL ***n = 100***	
	Number	***%***
RH Factor		
Negative	*0*	*0.0*
Positive	*100*	*100.0*

Supplementary Figure 1,2&3. Agarose gel electrophoresis for TNF genotypes from different exposure: ladder size marker (M) 50-1000 bp. **A** allele at 154bp, and **G** allele at 224bp. **(Supplementary)**

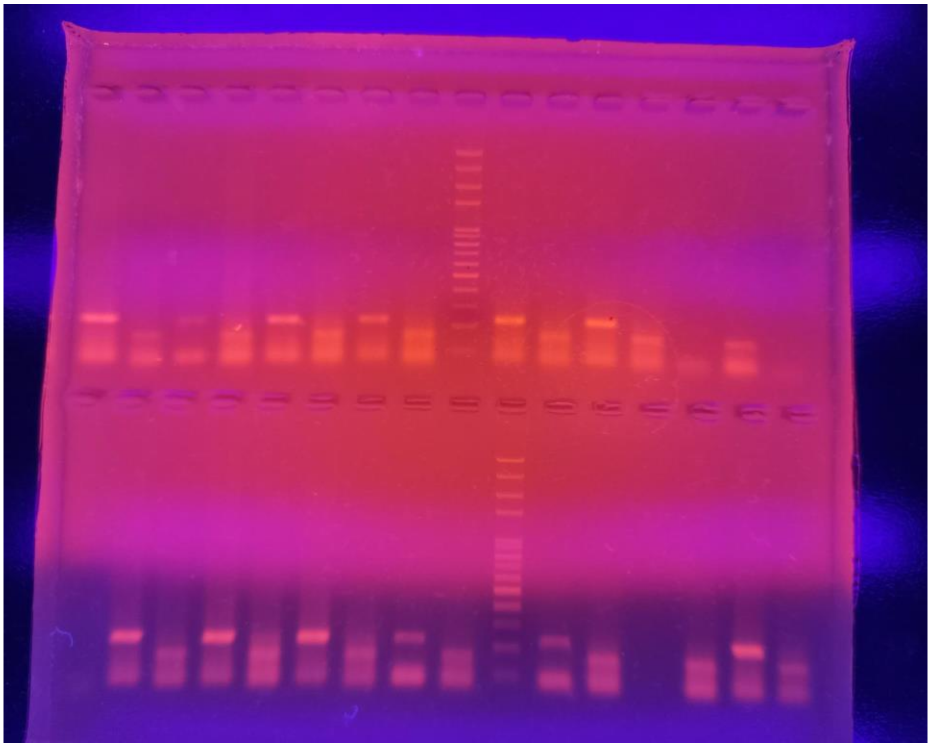

Supplementary Figure 1: S1

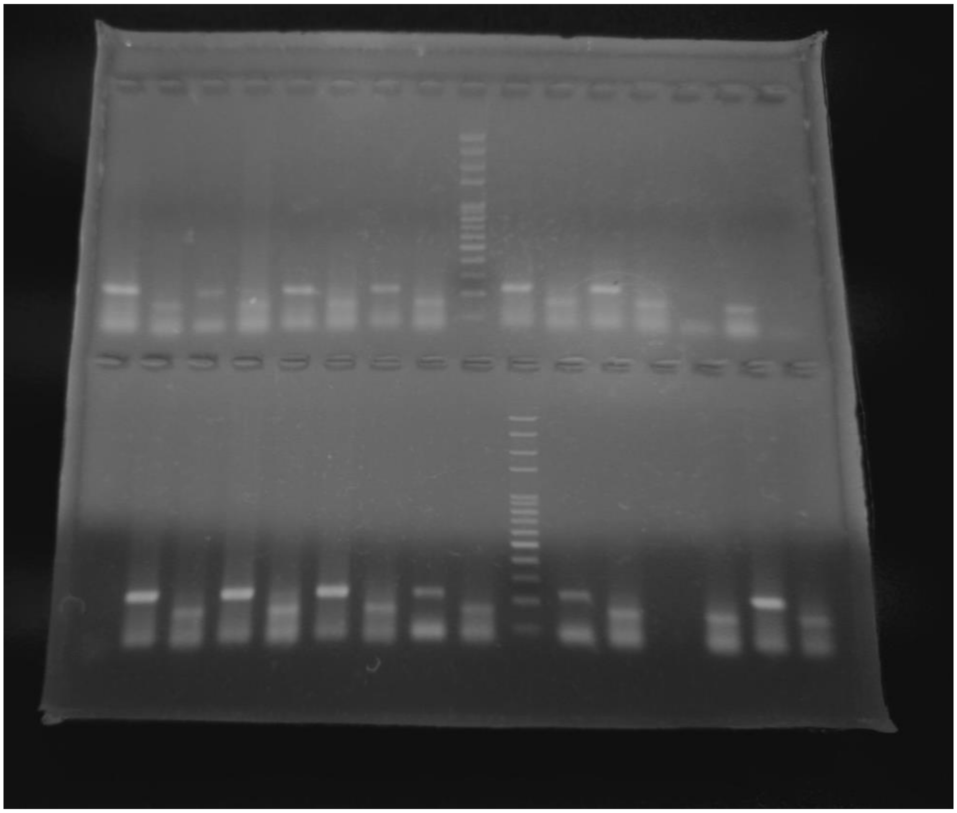

Supplementary Figure 2: S2

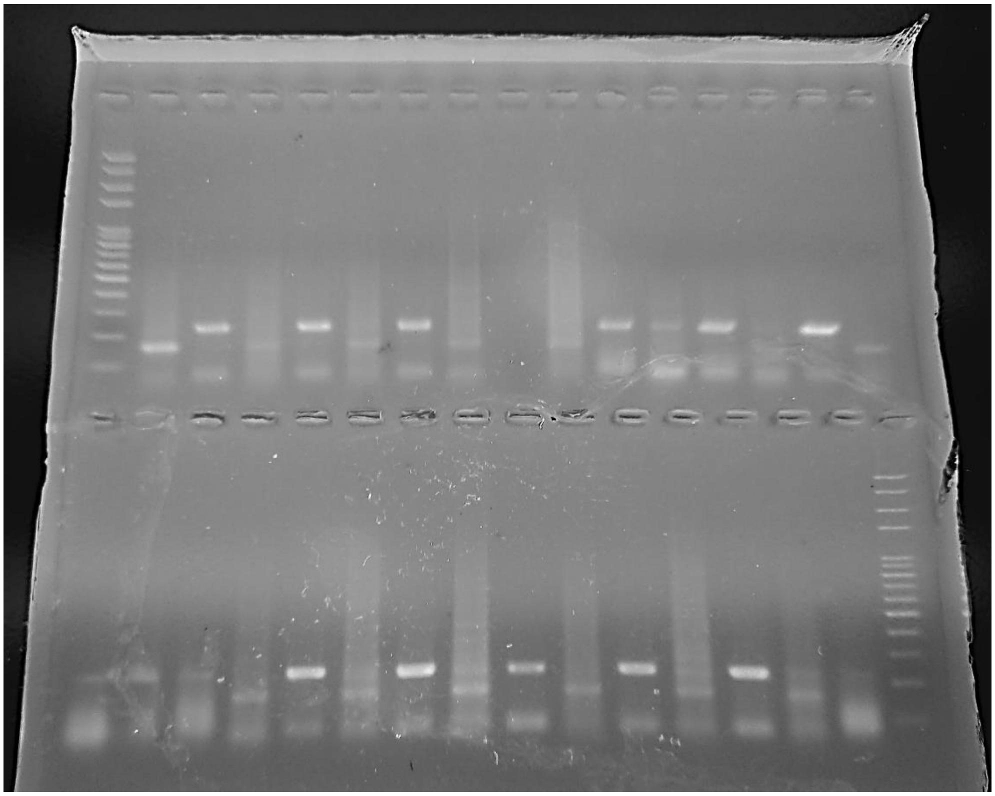

Supplementary Figure 3: S3

Supplementary Table 4:

S4. Association between TNFα −308 G/A (rs1800629) with weight loss among ALL patients.

	TNFα −308 G/A (rs1800629)						***X^2***	***p***
	GG n = 17		***GA n = 55***		***AA n = 28***			
Weight Loss								
Absent	15	88.2%	30	54.5%	16	57.1%	6.439	0.040
Present	2	11.8%	25	45.5%	12	42.9%		

X2, chi square test.

Supplementary Table 5:

S5. Association between TNFα −308 G/A (rs1800629) with laboratory data among ALL patients.

	TNFα −308 G/A (rs1800629)	***Test (p)***

	GG n = 17	***GA n = 55***	***AA n = 28***	
TLC (X10^9/L)				
Mean ± SD.	*11.55 ± 0.55*	*11.70 ± 0.74*	*11.42 ± 0.75*	*F= 1.379, p=0.257*
Median	*11.76*	*11.78*	*11.40*	
Range	*10.6 – 12.3*	*10.1 – 12.9*	*9.0 – 12.6*	
RBC (X10^12/L)				
Mean ± SD.	*3.81 ± 0.51*	*3.73 ± 0.51*	*3.92 ± 0.40*	*F= 1.503, p=0.228*
Median	*4.0*	*4.0*	*4.0*	
Range	*2.90 – 4.50*	*2.80 – 4.30*	*2.90 – 4.60*	
Hemoglobin (g/dL)				
Mean ± SD.	*8.66 ± 0.69*	*8.80 ± 0.75*	*8.70 ± 0.57*	*F= 0.345, p=0.709*
Median	*8.63*	*8.93*	*8.70*	
Range	*7.70 – 10.34*	*6.10 – 10.03*	*7.42 – 9.70*	
Platelet (X10^9/L)				
Mean ± SE.	*63.53 ± 3.61*	*84.18 ± 5.36*	*77.96 ± 6.04*	*H=2.843 p=0.241*
Median	*56.0*	*63.0*	*69.0*	
Range	*47.0 – 92.0*	*45.0 – 157.0*	*47.0 – 157.0*	
Peripheral blasts (%)				
Mean ± SD.	*27.06 ± 4.32*	*26.87 ± 4.59*	*26.75 ± 4.52*	*F= 0.025, p=0.976*
Median	*27.0*	*27.0*	*26.0*	
Range	*22.0 – 35.0*	*20.0 – 34.0*	*20.0 – 35.0*	
Bone marrow blasts (%)				
Mean ± SD.	*78.94 ± 19.24*	*84.0 ± 14.59*	*79.86 ± 15.52*	*F= 1.029, p=0.361*
Median	*82.0*	*89.0*	*83.0*	
Range	*44.0 – 101.0*	*46.0 – 105.0*	*45.0 – 97.0*	

SD, standard deviation, SE, standard error; min, minimum; max, maximum. F, ANOVA; H, Kruskal Wallis test. P: comparison between GG, GA and AA

Supplementary Table 6:

S6. Association between TNFα −308 G/A (rs1800629) with Immunophenotyping among ALL patients.

	TNFα −308 G/A (rs1800629)			***Test (p1)***
	GG ***n = 17***	***GA*** ***n = 55***	***AA*** ***n = 28***	
Immunophenotyping				
B-ALL	*16 (94.1%)*	*46 (83.6%)*	*20 (71.4%)*	*X2= 3.91, p=0.141*
T-ALL	*1 (5.9%)*	*9 (16.4%)*	*8 (28.6%)*	

X^2, chi square test.

IPT was not affected by rs1800629 genotypes (p>0.05).

Supplementary Figure 4. Column chart for association between TNFα −308 G/A (rs1800629) with FAB among ALL patients.

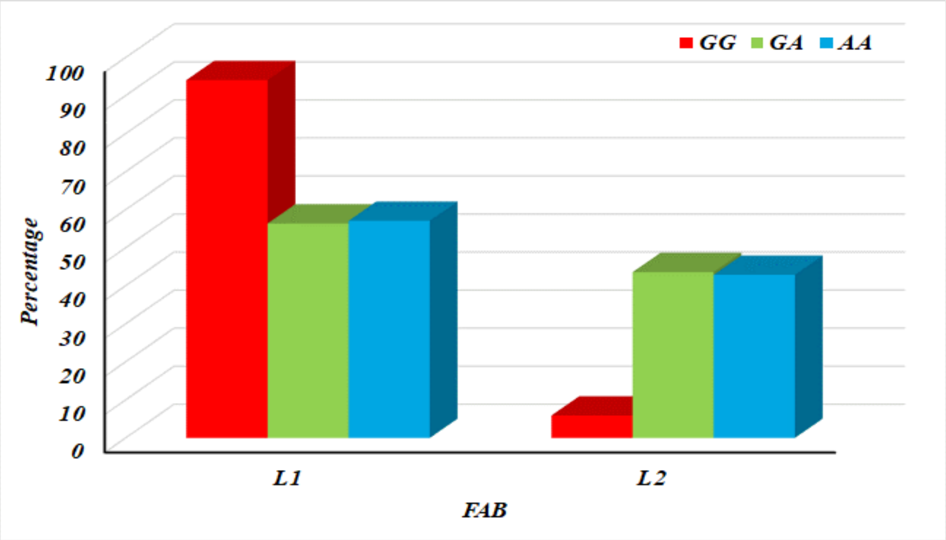

Supplementary Figure 4: S4

Supplementary Table 7:

S7. Association between TNFα −308 G/A (rs1800629) with TGFβ among control group.

	**TNFα −308 G/A (rs1800629)**			**p**
	GG	AG	AA	

Control	TGF-β (ng/mL)	Mean	73.96	81.63	69.25	0.167
		SE	2.20	3.48	7.49	
		Median	71	87	64	
		Minimum	44	21	58	
		Maximum	112	152	91	

Supplementary Table 8:

S8. Association between TNFα −308 G/A (rs1800629) with TGFβ among ALL group.

			**TNFα −308 G/A (rs1800629)**			***p***
			**GG**	**AG**	**AA**	
**ALL**	**TGF-β (ng/mL)**	Mean	25.68	17.85	16.81	**0.026**
		SE	3.08	1.57	2.25	
		Median	24	14	12	
		Minimum	8	5	5	
		Maximum	48	52	51	
